# Laboratory investigation into the role of largemouth bass virus (Ranavirus, Iridoviridae) in smallmouth bass mortality events in Pennsylvania rivers

**DOI:** 10.1186/s12917-018-1371-x

**Published:** 2018-03-02

**Authors:** Traimat Boonthai, Thomas P. Loch, Coja J. Yamashita, Geoffrey D. Smith, Andrew D. Winters, Matti Kiupel, Travis O. Brenden, Mohamed Faisal

**Affiliations:** 1 Department of Pathobiology and Diagnostic Investigation, College of Veterinary Medicine, Michigan State University, 1129 Farm Lane, Room 174, East Lansing, MI 48824 USA; 2 Division of Fish Production Services, Pennsylvania Fish and Boat Commission, State College, PA 16801 USA; 3 Division of Fisheries Management, Pennsylvania Fish and Boat Commission, Harrisburg, PA 17106 USA; 4 Comparative Medicine and Integrative Biology Program, College of Veterinary Medicine, Michigan State University, East Lansing, MI 48824 USA; 5 Department of Fisheries and Wildlife, College of Agriculture and Natural Resources, Michigan State University, East Lansing, MI 48824 USA; 6 Present address: Department of Microbiology, Immunology, and Biochemistry, School of Medicine, Wayne State University, Detroit, MI 48201 USA

**Keywords:** Largemouth bass virus, Smallmouth bass, *Flavobacterium columnare*, *Aeromonas salmonicida*, Susquehanna River basin, Dermal lesions

## Abstract

**Background:**

Mortality episodes have affected young-of-year smallmouth bass (*Micropterus dolomieu*) in several river systems in Pennsylvania since 2005. A series of laboratory experiments were performed to determine the potential role of largemouth bass virus (*Ranavirus*, Iridoviridae) in causing these events.

**Results:**

Juvenile smallmouth bass experimentally infected with the largemouth bass virus exhibited internal and external clinical signs and mortality consistent with those observed during die-offs. Microscopically, infected fish developed multifocal necrosis in the mesenteric fat, liver, spleen and kidneys. Fish challenged by immersion also developed severe ulcerative dermatitis and necrotizing myositis and rarely panuveitis and keratitis. Largemouth bass virus-challenged smallmouth bass experienced greater mortality at 28 °C than at 23 or 11 °C. Co-infection with *Flavobacterium columnare* at 28 °C resulted in significant increase in mortality of smallmouth bass previously infected with largemouth bass virus. *Aeromonas salmonicida* seems to be very pathogenic to fish at water temperatures < 23 °C. While co-infection of smallmouth bass by both *A. salmonicida* and largemouth bass virus can be devastating to juvenile smallmouth bass, the optimal temperatures of each pathogen are 7–10 °C apart, making their synergistic effects highly unlikely under field conditions.

**Conclusions:**

The sum of our data generated in this study suggests that largemouth bass virus can be the causative agent of young-of-year smallmouth bass mortality episodes observed at relatively high water temperature.

## Background

Since 2005, mortality episodes of young of the year (YOY) smallmouth bass (*Micropterus dolomieu*; SMB) have been consistently reported from a number of river systems in Pennsylvania, including the Susquehanna, Juniata, and Allegheny Rivers. These mortality events have been more persistent and severe in the Susquehanna and Juniata River systems, occurring annually to varying degrees, than in the Allegheny and other drainages. These SMB die-offs have created considerable concerns among the sport-fishing industry, as well as in state and federal agencies, as decreases in relative abundance of YOY and adult SMB and shifts in size structure have concurrently been noticed [1]. Affected SMB exhibited exophthalmia, dermal lesions (e.g., fin erosions and rounded, shallow ulcers), and organomegaly. The prevalence of moribund fish varied both spatially and temporally and was most prevalent during years with high water temperatures [1]. Several fish-pathogenic bacteria and parasites were reported from fish collected during the course of mortality episode examinations, such as *Aeromonas* spp., *Shewanella putrefaciens, Flavobacterium columnare*, *Pseudomonas aeruginosa*, myxozoa (e.g., *Myxobolus inornatus*) and trematodes [1–4]. Largemouth bass virus (LMBV, genus *Ranavirus*, Family Iridoviridae) has also been isolated from both apparently healthy and moribund SMB during fish-kill episodes in several river watersheds in Pennsylvania (4, https://www.fws.gov/wildfishsurvey/) and the Chesapeake Bay watershed [5]. The contributions of each of these pathogens (single or combined) in causing these recurrent SMB kills have not been thoroughly investigated under controlled laboratory conditions.

Most of the existing knowledge on LMBV is derived from surveys and experiments performed on largemouth bass (*Micropterus salmoides*; LMB), although this virus has been isolated from other centrarchid species. Typically, LMBV-infected LMB exhibit lethargy, external and internal hemorrhages, and organomegaly [6, 7]. However, Deng et al. [8] described an outbreak in LMB in China that was associated with the formation of widespread ulcerative lesions. The causative agent was a ranavirus named largemouth bass ulcerative syndrome virus (LBUSV) by Deng et al. [8]. Upon phylogenetic analysis of LBUSV major capsid protein gene (MCP), it proved to be a ranavirus sharing 100% similarity to the Doctor Fish Virus (DFV) and 98% similarity to LMBV. Most recently, another ranavirus, named LMBV-like, was detected in farmed barcoo grunter (*Scrotum barcoo*) in Thailand during outbreaks that were associated with ulcerative lesions [9]. The MCP gene of Thai ranavirus shared 100% similarity to the Chinese LBUSV, clustered in the same clade with high bootstrap support, and shared 99.3–99.5% similarity to LMBV and DFV.

This study was designed to investigate the role LMBV may play in causing the recurrent YOY SMB mortality episodes. First, we screened five LMBV isolates that were associated with YOY SMB mortality episodes for their pathogenicity to SMB using IP and immersion infection methods. Second, we described gross clinical signs and histopathological alterations of LMBV-experimentally infected fish. Last, we determined the role that two other fish-pathogenic bacteria may play in exacerbating LMBV infection. The series of experiments described herein unravel some of the potential mechanisms leading to the YOY SMB mortality episodes noticed in several watersheds in Pennsylvania.

## Methods

### Fish

All protocols described in this study involving the use of live fish were approved by the Michigan State University (MSU) Institutional Animal Care and Use Committee (IACUC AUF # 08/15–129-00). Health-certified YOY SMB (fork length: 9.7 ± SD 2.1 cm; weight: 11.2 ± SD 6.3 g) were obtained from Zetts Fish Farm and Hatchery (Drifting, PA), and the New Jersey Department of Environmental Protection Division of Fish and Wildlife. Upon arrival at the MSU-Research Containment Facility, fish were allowed to acclimate to laboratory conditions in 720-L rectangular fiberglass tanks at a water temperature of 22 ± 1 °C for 4 weeks. Fish were fed commercial pellets (Classic Fry – 1.5 mm, Skretting Co., Tooele, UT) and tanks were cleaned daily. A subsample of 10 SMB was screened for the presence of LMBV and other viruses in their visceral organs using the fathead minnow (FHM) cell line as detailed below.

### Cell lines and LMBV propagation

FHM cell line was grown and maintained in 150 cm^2^ tissue culture flasks (Corning, Inc., Corning, NY) at 25 °C using growth formulation of Hanks’ balanced salts minimum essential medium (Invitrogen, Carlsbad, CA) supplemented with 10% fetal bovine serum (GemCell, West Sacramento, CA), 2.0 mM L-glutamine (Invitrogen), penicillin (100 U/ml)/streptomycin (100 μg/ml; Gibco, Grand Island, NY) and amphotericin B (2.5 μg/ml; BioWhittaker, Walkersville, MD).

**Fig. 1:**
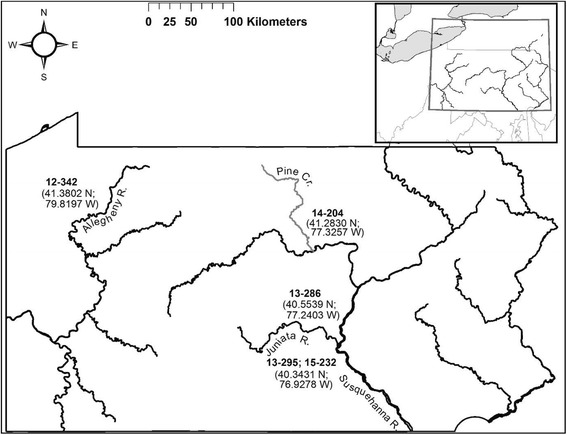
Geographic origin of the five largemouth bass virus (LMBV) strains used in this study. The designation of the LMBV isolate recovered from moribund fish are indicated in bold, and the latitude and longitude of the smallmouth bass (SMB; *Micropterus dolomieu*) mortality episodes are listed in parentheses

In this study, we used five LMBV isolates that were isolated from YOY SMB during mortality episodes in the Susquehanna and Allegheny (the principal tributary of the Ohio River) river systems that were designated 13–295 Susquehanna and 15–232 Susquehanna (both isolated from SMB from the lower Susquehanna River sub-basin), 13–286 Juniata and 14–204 Pine Creek (a creek in the West Susquehanna River sub-basin), and 12–342 Allegheny (Fig. 1). These LMBV isolates were originally isolated by the U.S. Fish and Wildlife Northeast Fishery Center, Lamar, PA and Pennsylvania State Animal Health Diagnostic Laboratory, State College, PA and were made available for this study by the Pennsylvania Fish and Boat Commission. Virus stocks were produced using FHM cells grown in 150 cm^2^ tissue culture flasks and incubated at 28 °C. To determine the virus concentration, a median tissue culture infectious dose (TCID_50_) was determined using FHM cells and calculations of virus titers were done as described by Reed and Muench [10]. The virus stocks were aliquoted in cryogenic vials (Corning, Inc.) and stored at − 80 °C until used.

### Experimental infection of SMB by Pennsylvania LMBV isolates

Two studies were performed. The first experiment was to determine if the five LMBV isolates could induce clinical signs and histopathological changes in juvenile SMB. Twenty groups of fish (*n* = 5/tank/dose) were housed in 70-L static circular tanks equipped with air-driven sponge filters (Hydro–Sponge III Filter, Aquarium Technologies, Decatur, GA). Water temperature was elevated by 2 °C/day to reach 28 °C ± 1 °C, which is reported to be the optimum temperature for LMBV experimental infection [11], using submersible 150-W heaters (Aqueon®-Pro 150, Central Aquatics™, Franklin, WI). Prior to injection, fish were anesthetized with sodium bicarbonate-buffered tricaine methanesulfonate (100 mg/L; MS-222, Western Chemical, Ferndale, WA). Fish were IP injected with 50 μl of tissue culture medium (TCM) containing LMBV at one of four concentrations: 10^1^, 10^3^, 10^5^ and 10^7^ TCID_50_/fish. The negative control consisted of fish (*n* = 5) that were sham-injected with 50 μl of LMBV-free TCM. Fish were monitored daily for 30 days, whereby disease signs, cumulative mortality and behavior were recorded daily.

Based on LMBV intraperitoneal (IP) experimental infection results, the 13–286 Juniata and 13–295 Susquehanna LMBV isolates were selected for SMB waterborne challenge. Immersion exposures (*n* = 10 fish/tank/dose) were performed in aerated water at six final concentrations (10^1^, 10^2^, 10^3^, 10^4^, 10^6^ and 10^7^ TCID_50_/ml) for each of the two LMBV isolates for 1 h and then placed back into their respective tanks. An additional group of fish (n = 10/tank) was subjected to same procedure except that they were immersed in water containing a LMBV-free TCM and were considered negative controls. Fish were kept at 28 ± 1 °C in circular 70-L tanks and clinical signs and mortality were recorded daily. Results of the waterborne experimental infection facilitated estimating a dose of LMBV that would allow the survival of some infected fish after a waterborne encounter with the virus.

### Sample collection for LMBV-re-isolation and histopathology

Moribund fish were euthanized with an overdose (250 mg/L) of MS-222. At the end of the observation period for each experiment, all surviving fish were euthanized. Freshly dead and moribund fish, as well as fish that were euthanized at the end of the observation period, were necropsied. Portions of kidneys, spleen, and liver were collected in 1.5-ml centrifuge tubes and kept frozen at − 80 °C until processed for LMBV re-isolation and confirmation.

To examine tissue pathological consequences associated with LMBV infection in SMB, portions of external and internal lesions of each of LMBV-infected fish (moribund and recently dead from IP and water challenge experiments) were collected and fixed in 10% buffered formalin. Fixed samples were then dehydrated in a graded series of alcohol, embedded in paraffin, sectioned and stained with hematoxylin and eosin [12]. Stained tissues were visually examined under a light microscope (Olympus, Model BX41TF, Kyoto, Japan) and photographs were taken using image software (Olympus DP25-BSW, version 2.2) connected to a camera (Olympus DP25).

### Molecular characterization of LMBV

Viral DNA from cell culture was purified using a DNeasy® Blood & Tissue Kit (Qiagen, Hilden, Germany) according to the manufacturer’s instructions. The full-length major capsid protein gene sequences were PCR amplified using the forward 5′- ATG TCT TCT GTT ACG GGT TCT GGC-3′ and reverse 5′- TTA CAG GAT GGG GAA ACC CAT GG-3′ primer pairs and sequenced. Each PCR reaction contained 12.5 μl of GoTaq Green Master Mix (Promega, Madison, WI), 0.8 μM of each primer, 70 ng of DNA template and DNAase-free water to produce a final volume of 25 μl. Amplification was performed in a GeneAmp® PCR System 9700 thermocycler (AB Applied BioSystems, Foster City, CA) using a single denaturation step at 94 °C for 5 min, followed by 30 cycles of 94 °C for 30 s, 55.0 °C for 30 s and 72 °C for 1.5 min, with an additional elongation at 72 °C for 7 min. The expected amplicon (1392 bp) was visualized in 1.5% (*w*/*v*) agarose gel electrophoresis containing SYBR safe DNA gel stain (Invitrogen, Carlsbad, CA) for 30 min at 100 V under UV transillumination (UVP, Model TFM-26, Upland, CA). The resulting sequences were submitted for basic local alignment search tool analysis (BLAST) [13] against the nr database for sequence similarity searches. The resulting sequences (1392 bp) were deposited in GenBank (Accession #: KY825779-KY825781).

### Effect of temperature on SMB susceptibility to LMBV

The effect of temperature on SMB susceptibility to LMBV was evaluated using the 13–286 Juniata LMBV isolate. Water temperature was adjusted to one of three temperatures: 11 ± 1, 23 ± 1 and 28 ± 1 °C (two tanks/temperature). Fish (*n* = 10/tank/treatment) were randomly chosen for each tank and allowed to acclimate for 7 days. Fish for each of the temperatures were infected with LMBV at a concentration of 10^2.6^ TCID_50_/ml in 10-L glass aquaria by immersion for 60 min. Fish in temperature-matched control (three tanks) were immersed in 10-L of diluted sterile TCM. Experimental fish were observed for morbidity and mortality for 30 days and samples were collected as described above.

### Role of concurrent infection with *F. columnare* or *Aeromonas salmonicida* on SMB LMBV mortality

*Flavobacterium columnare* strain 10FC isolated in the authors’ laboratory in 2014 and *Aeromonas salmonicida* subspecies *salmonicida* strain 3.205 isolated from SMB during a mass mortality event in 2011 at the southern fork of the Shenandoah River, Millville, WV and provided by Dr. Rocco Cipriano (the US Geologic Survey National Fish Health Center, Leetown, WV) were used in experimental co-infection with LMBV to determine the combined infection effects on fish. All infections were performed via the waterborne immersion route.

For co-infection trials of the 13–286 Juniata LMBV strain and *F. columnare,* 12 tanks (8 SMB/tank), divided into four treatments (3 replicates per treatment), were used. Fish in the first treatment were exposed to neither virus nor bacterium and diluent vehicle was added to their tanks (10-ml TCM/tank added at day 1 and 10-ml phosphate buffered saline [PBS])/tank added at day 10) and were considered a double negative control group. Fish in the second treatment were immersed in suspension of 13–286 Juniata LMBV strain at a concentration of 10^2.6^ TCID_50_/ml for 1 h on day 1, and on day 10, 10-ml PBS/tank was added into the water. This treatment was considered the LMBV positive group. In the third treatment, fish were held in water with TCM for 10 days before exposing fish to *F. columnare* at a final concentration of 1.75 × 10^8^ CFU/ml in glass tanks at day 10. The fourth treatment received both LMBV at day 1 and *F. columnare* at day 10 at the same concentrations and conditions described above. Morbidity and mortality of SMB were monitored for 30 days.

The co-infection study with *A. salmonicida* followed the same design, except that following LMBV infection, the water temperature was dropped from 28 to 20 °C over three days, since the optimal temperature for *A. salmonicida* infection ranges from 15 to 20 °C [14]. The bacterial dose used for infection was 1.35 × 10^8^ CFU/ml collected during the logarithmic phase of in vitro growth.

For re-isolation of *F. columnare* 10FC and *A. salmonicida* 3.205 in co-infection trials, kidney tissues and external lesions were collected from dead, moribund and surviving fish that were euthanized using inoculating loops and streaked onto Hsu-Shotts medium supplemented with neomycin sulphate (4 mg/L) for *F. columnare* [15] and Trypticase Soy Agar for *A. salmonicida* [16]. All plates were incubated at 22 °C for up to 72 h. Individual representative colonies were subcultured and confirmed by molecular analyses (below).

### Virus and bacteria re-isolation and confirmation

LMBV re-isolation was performed using FHM cells following USFWS and AFS-FHS [16] protocol. Tissue pools (kidney, spleen and liver) were diluted 1:20 (*w*/*v*) in Eagle’s minimum essential medium (Gibco) supplemented with 0.3% tryptose phosphate broth (BD Biosciences, Sparks, NY), penicillin (100 U/ml), streptomycin (100 μg/ml), and amphotericin B (2.5 μg/ml) and then homogenized using a hand-held pestle and tissue grinder (Fisherbrand®, Fisher Scientific, Pittsburgh, PA) in a 1.5-ml centrifuge tube. Samples were clarified by centrifugation at 5000 rpm (2711×*g*) for 30 min. Supernatants were inoculated (25 μl) in triplicate wells of 96-well microplates containing s monolayer of FHM cells. Infected plates were incubated at 28 °C and observed for the formation of cytopathic effect (CPE; i.e., cell lysis with wide areas of monolayer detachment). After 7 days post-incubation, cultures with no evidence of CPE were blind passaged by inoculating fresh FHM cells with cell culture supernatant and observed for an additional 7 days. Samples that exhibited the formation of CPE at any time during incubation were considered presumptively positive for LMBV. Confirmatory identification of the virus from cell culture supernatant was made by polymerase chain reaction (PCR) as described below.

To confirm that the virus isolated from experimentally infected fish was LMBV, total DNA was extracted from CPE-positive cell culture using a DNeasy® Blood & Tissue Kit following the manufacturer’s protocol and then stored at − 20 °C. For samples that exhibited no signs of CPE at the end of the second passage, DNA extraction was performed directly from collected tissues using the same extraction kit. Extracted DNA was quantified using a Qubit fluorometer (Invitrogen, Eugene, OR). Primer pairs used to confirm LMBV were LMBV288F: 5’-GCG GCC AAC CAG TTT AAC GCA A -3′ and LMBV353R: 5′- AGG ACC CTA GCT CCT GCT TGA T -3′ targeting a portion of LMBV major capsid protein [17]. Each PCR reaction contained 12.5 μl of GoTaq Green Master Mix (Promega, Madison, WI), 0.8 μM of each primer, 70 ng of DNA template and DNAase-free water to produce a final volume of 25 μl. Amplification was performed in a GeneAmp® PCR System 9700 thermocycler (AB Applied BioSystems) using a single denaturation step at 94 °C for 5 min, followed by 30 cycles of 94 °C for 30 s, 56.5 °C for 30 s and 72 °C for 35 s, with an additional elongation at 72 °C for 7 min. The expected amplicon (248 bp) was visualized in 1.5% (*w*/*v*) agarose gel electrophoresis containing SYBR safe DNA gel stain (Invitrogen) for 30 min at 100 V under UV transillumination (UVP, Model TFM-26, Upland, CA).

DNA was extracted from re-isolated bacteria using a DNeasy® Blood & Tissue Kit according to the manufacturer’s protocol. Bacterial DNA was quantified as previously described. PCR amplification of *F. columnare* was conducted using the primers FCISRFL (5′ -TGC GGC TGG ATC ACC TCC TTT CTA GAG ACA - 3′) and FCISRR1 (5′ -TAA TYR CTA AAG ATG TTC TTT CTA CTT GTT TG – 3′; detailed in Faisal et al. [18]). To confirm *A. salmonicida*, amplified PCR product was produced using MIY1 (5′- AGC CTC CAC GCG CTC ACA GC- 3′) and MIY2 (5′- AAG AGG CCC CAT AGT GTG GG -3′) primers (detailed in [19]).

### Statistical analysis

For examining the effect of temperature on SMB susceptibility to LMBV, differences in Kaplan-Meier survival estimates between LMBV and control treatments were tested with log-rank tests at each experimental temperature. Additionally, differences in overall cumulative mortality between LMBV and control treatments at each experimental temperature were tested using Fisher’s exact test for proportions.

For the co-infection experiments, we initially tested for differences in Kaplan-Meier survival estimates among the replicate tanks within each treatment with log-rank tests. If statistically significant differences among the replicates were not detected, we combined the replicate tank results for each treatment and tested for an overall difference in Kaplan-Meier survival estimates among the treatments with a log-rank test. If the overall test was statistically significant, we then conducted pairwise log-rank tests between the infection treatments. Bonferroni corrections were applied to protect the error-rate of the pairwise tests. Differences in overall cumulative mortality among the treatments were tested using a mixed-effects logistic regression model with treatment as a fixed effect and tank as a random effect. Pairwise differences in infection treatments were then tested if there were overall statistically differences among the treatments. As with the pairwise log-rank tests, Bonferroni corrections were applied to protect the error-rate of the pairwise tests. Kaplan-Meier survival curves and log-rank tests were conducted in R [20] using the survive [21] and survminer [22] libraries. Fisher’s exact tests and mixed-effect logistic regression models were conducted in SAS Version 9.4 (SAS Institute Inc., Cary, NC) using PROC FREQ and PROC GLIMMIX, respectively [23].

## Results

### Exposure of SMB to LMBV

Exposure of SMB to LMBV by IP resulted in morbidity and mortality in all LMBV infected groups with variable rates, the 13–295 Susquehanna LMBV and the 13–286 Juniata isolates being the most pathogenic to SMB. Mortality in SMB in the groups that received LMBV at a concentration 10^3^–10^7^ TCID_50_/fish started at the 2^nd^ day post-infection (pi) and reached a cumulative mortality of 100% within 14 days pi, except the fish exposed to 12–342 Allegheny and 14–204 Pine Creek strains. In the groups challenged with 10^1^ TCID_50_/fish, mortality was observed only in 13–295 Susquehanna and 13–286 Juniata infections, reaching a cumulative mortality of 60 and 40%, respectively.

**Fig. 2:**
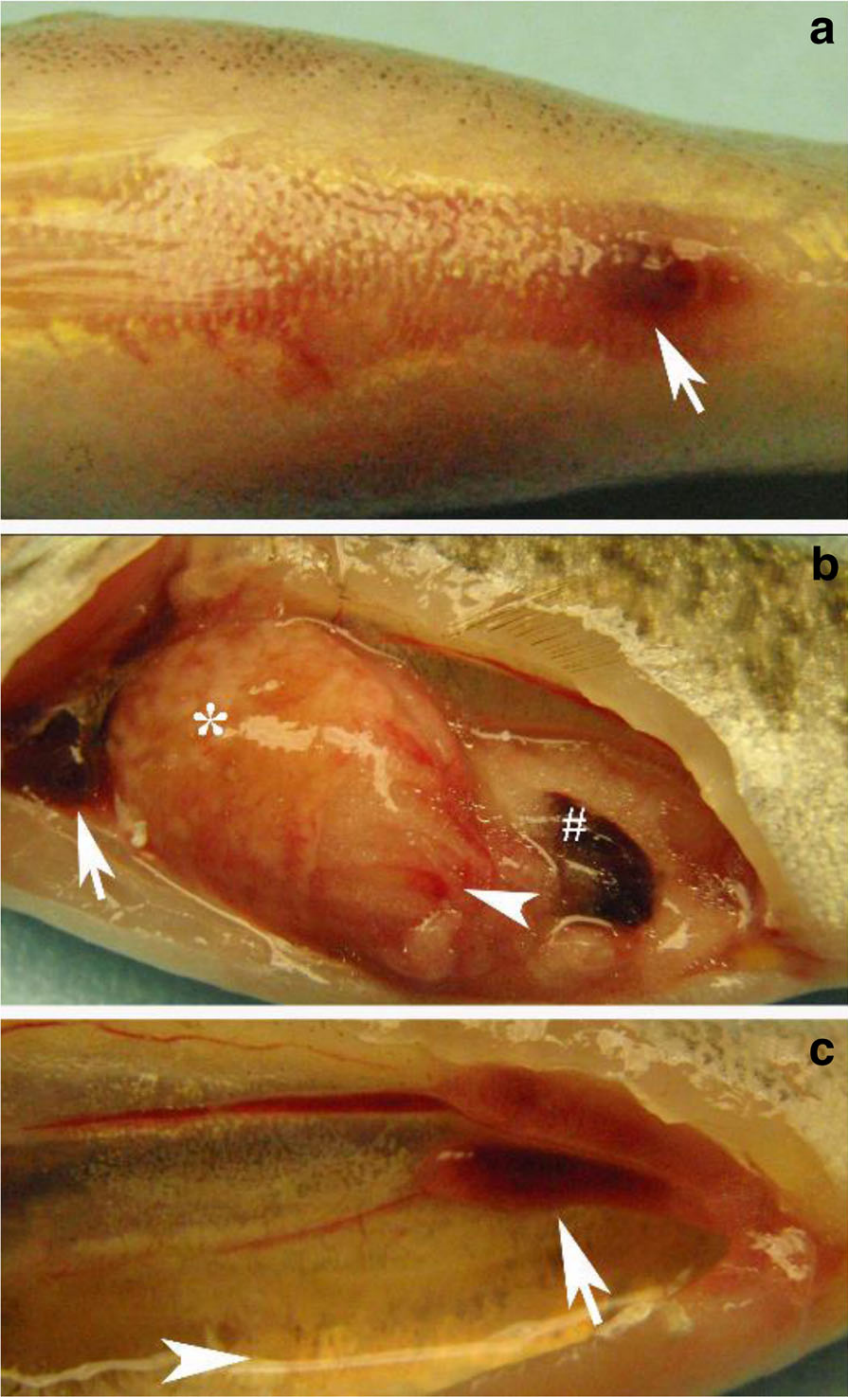
Clinical signs in largemouth bass virus (LMBV)-IP injected smallmouth bass (SMB; *Micropterus dolomieu*). **a** abdominal distension along with subdermal hemorrhage on ventral area and vent (arrow) of 13–295 Susquehanna-injected SMB; **b** swelling and hemorrhage on heart ventricle (arrow) concomitant with hemorrhage of visceral fat (arrowhead), mottled, friable, edematous and enlarged livers (*) and enlargement with darkening and edema of spleen (#) of SMB exposed to 13–286 Juniata; and **c** severe ascites (arrowhead) and ecchymotic hemorrhage in gonads of 12–342 Allegheny challenged SMB

Moribund SMB were lethargic and some displayed gill pallor, abdominal distension, hemorrhages on ventral area, and hemorrhagic protruded vents (Fig. 2a). In some fish, intestinal prolapse, and petechial to diffuse hemorrhages on opercula, isthmus, mandible and maxilla were observed. Visceral organs appeared edematous with hemorrhages of various sizes on visceral fat (Fig. 2b) and heart ventricles (Fig. 2b). Intestines appeared inflamed and contained clear mucus content, while livers were mottled and enlarged with hemorrhages of various sizes (Fig. 2b). Splenomegaly, petechial to ecchymotic hemorrhage within the swim bladder, and diffuse hemorrhage within the gonads (Fig. 2c) were observed. Kidneys were enlarged with hemorrhages within the tissues. LMBV was isolated from all dead and surviving SMB and its identity confirmed by conventional PCR. Mock-infected fish showed no clinical signs and no virus was detected in their tissues.

**Fig. 3:**
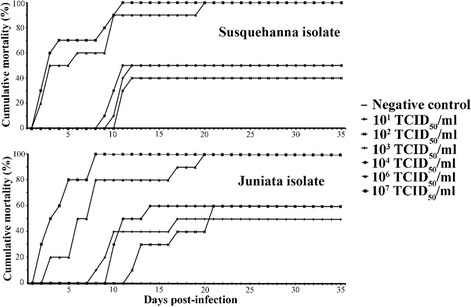
Cumulative mortality of largemouth bass virus (LMBV) in smallmouth bass (*Micropterus dolomieu*). Fish were experimentally infected with two LMBV strains by immersion

Based on the observations made during the IP infection screening study, the 13–295 Susquehanna and 13–286 Juniata isolates were selected for infecting SMB by immersion. The two LMBV isolates induced mortalities in all challenged SMB at all doses examined, except for dose of 10^1^ TCID_50_/ml (Fig. 3). Cumulative mortality of 13–295 Susquehanna-infected SMB in the groups that received the low virus doses (10^2^, 10^3^ and 10^4^ TCID_50_/ml) reached 40–50% within 9 and 12 days pi, while mortality in SMB in the high dose groups (10^6^ and 10^7^ TCID_50_/ml) began at 2 days pi and continued until 20 days pi, reaching a cumulative mortality of 100% (Fig. 3). Mortality of Juniata-challenged SMB was comparatively more severe than that of the Susquehanna isolate. In the low dose groups (10^2^, 10^3^ and 10^4^ TCID_50_/ml), mortality started between 8 and 12 d pi and peaked to 50–60% by the 21^st^ day pi. In SMB exposed to the high doses (10^6^ and 10^7^ TCID_50_/ml), mortality started as early as 2^nd^ day pi and continued up to 20 days pi to reach a cumulative value of 100% (Fig. 3).

**Fig. 4:**
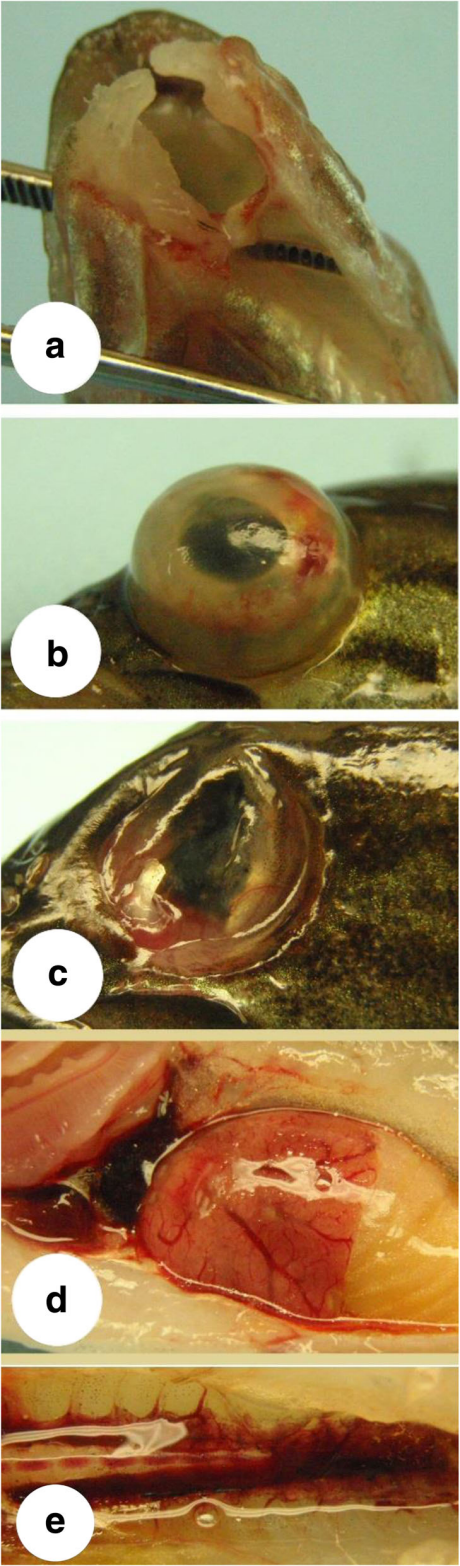
Clinical signs observed in smallmouth bass (*Micropterus dolomieu*) infected with largemouth bass virus (LMBV) by immersion. **a** broken mandible at the middle with ulceration and hemorrhage in a fish infected with the 13–295 Susquehanna River LMBV isolate; **b** unilateral exophthalmia with the protruding eye ball concurrent with ulceration, intraocular hemorrhage and corneal opacity in a fish infected with the 13–286 Juniata River LMBV isolate, and **c** ruptured eye showing loss of vitreous, lenticular damage and periocular hemorrhage commonly seen in an smallmouth bass that survived infection with the 13–286 Juniata River LMBV isolate. **d** gill pallor, hemorrhagic heart ventricle, severe ascites, ecchymotic hemorrhage with enlargement and edema on liver of 13–286 Juniata LMBV-infected fish; and **e** edematous, friable and ecchymotic hemorrhage observed in kidneys of 13–295 Susquehanna LMBV-infected fish

**Fig. 5:**
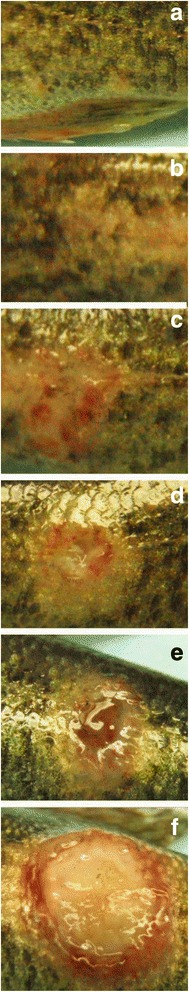
Progression of dermal lesions in largemouth bass virus-challenged smallmouth bass (*Micropterus dolomieu*). **a** Faint skin discoloration on the trunk. **b** Further progressed skin discoloration. **c** Skin discoloration accompanied by hemorrhage and early stage of scale loss. **d** Skin discoloration surrounding a shallow dermal ulcer with hemorrhagic margins and central scale loss. **e** Deeper ulcer penetrating into the underlying muscular layer with surrounding skin discoloration. **f** Severe ulceration with well-circumscribed, raised, and hemorrhagic edges

Gross clinical signs in LMBV-infected SMB by immersion were, to a great extent, similar to those observed in IP-infected fish. In addition, there were some lesions in the two immersion groups that were not observed in the IP infected SMB groups. For examples, ~ 25% of SMB exhibited signs of emaciation, disjointed mandibles (Fig. 4a), uni- or bilateral exophthalmia associated with ecchymotic hemorrhages and corneal opacity (Fig. 4b) that can lead to a rupture of the eye, loss of the vitreous, and subsequent collapse (Fig. 4c) in some survivors. Unique to the immersion group was the presence of external ulcers that began as discoloration of the skin (Fig. 5a) that expanded in size (Fig. 5b) and eventually was accomplanied by hemorrhage and scale loss (Fig. 5c, d). These lesions then progressed to ulcers (0.5-3 cm in diameter) with white centers and hemorrhagic well circumscribed edges that eventually penetrated into the underlying muscular layer (Fig. 5d-f). Moreover, some infected SMB exhibited eroded and ulcerated fins. Internal organs showed lesions that were similar to those observed with the IP infected group; namely, ascites, hemorrhagic visceral fat, enlarged hemorrhagic heart ventricles (Fig. 4d); hemorrhagic, enlarged liver (Fig. 4d), and darkened, friable and enlarged spleens. Hemorrhages within the gonads, and enlarged, hemorrhagic kidneys were also observed (Fig. 4e).

### Virus re-isolation and histopathology

LMBV was isolated from all moribund and surviving fish from all dose groups (except the lowest dose group) of all isolates and confirmed by PCR. Neither gross clinical signs were observed, nor was LMBV isolated or detected by PCR from mock-challenged SMB.

**Fig. 6:**
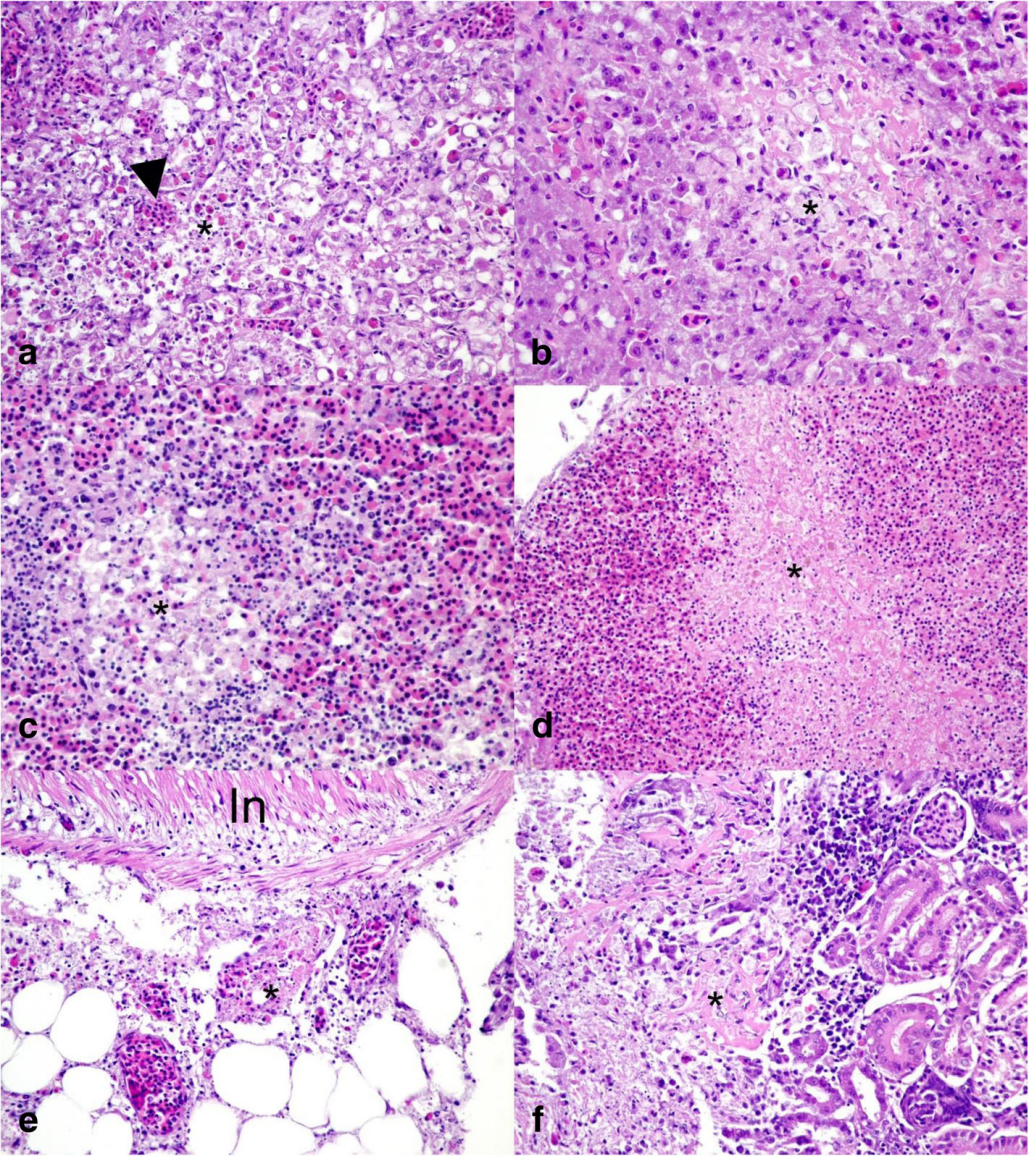
Histopathological lesions in smallmouth bass (*Micropterus dolomieu*) infected with the largemouth bass virus. **a** Focally extensive hepatocellular degeneration and necrosis (asterisk) with secondary infiltration of heterophils (arrowhead). **b** Liver with focal area of coagulative necrosis (asterisk) characterized by loss of nuclear detail and hypereosinophilia of affected hepatocytes surrounded by cellular debris and few infiltrating heterophils. **c** Focal splenic necrosis (asterisk) with lymphocytolysis and karyorhectic debris. **d** Spleen with confluent area of coagulative necrosis (asterisk). **e** Mesenteric fat with focally extensive necrotizing steatis (asterisk) adjacent to the intestinal wall. **f** Kidney with focal area of fibrinoid necrosis (asterisk) with loss renal tubules and replacement by accumulating fibrin admixed with cellular debris and macrophages. Fish in **a**, **c**, and **e** were infected by intraperitoneal injection, while fish in **b**, **d**, and **f** were infected by immersion

**Fig. 7:**
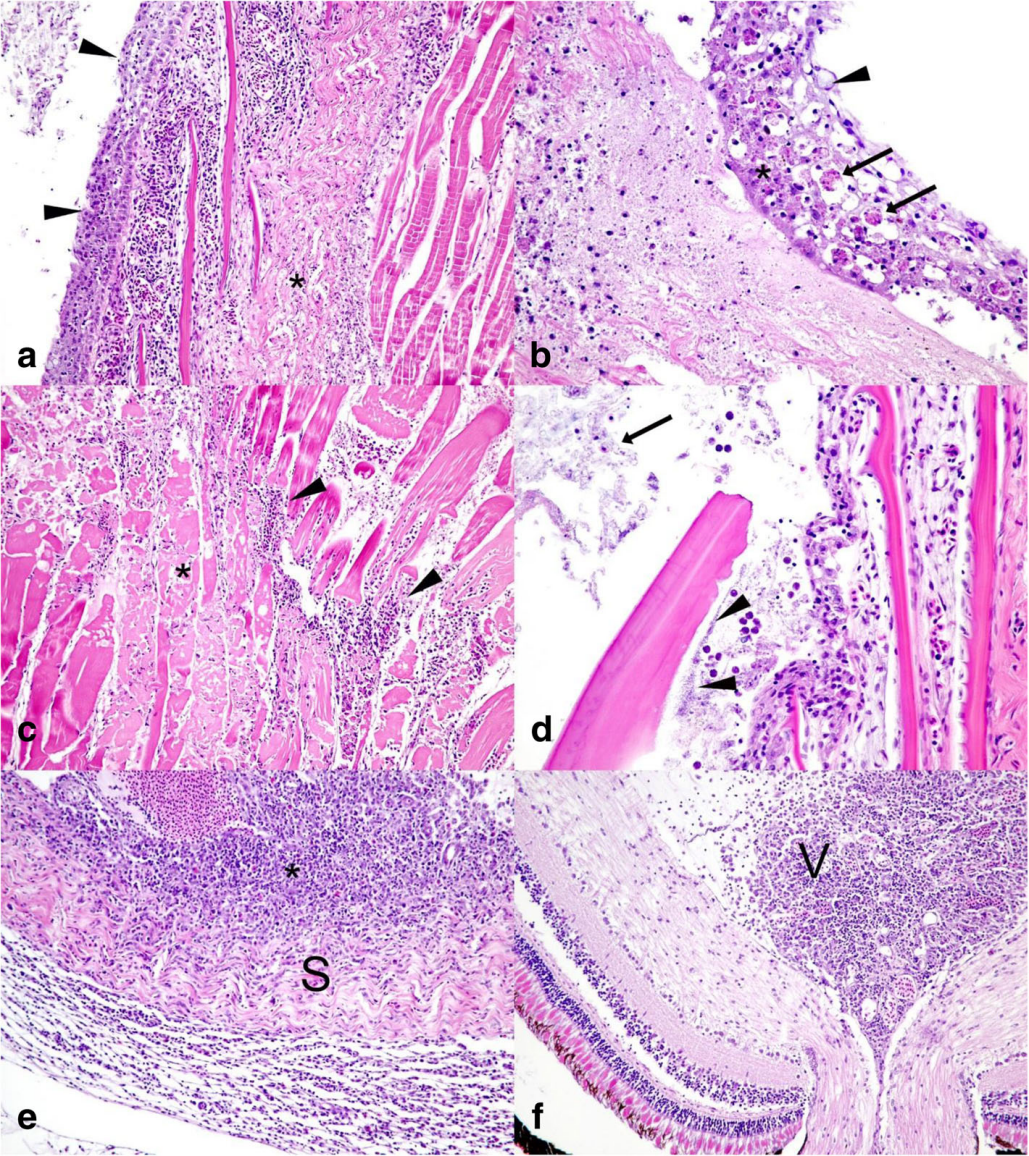
Histopathological lesions in smallmouth bass (*Micropterus dolomieu*) infected with largemouth bass virus. **a** Extensive ulcerative dermatitis (arrowheads) with severe epithelial necrosis (asterisk). **b** Higher magnification of the affected epithelium with cytoplasmic swelling and vacuolation of epithelial cells (arrowhead), nuclear pyknosis (asterisk) and infiltration of heterophils (arrows). **c** Severe necrotizing myositis with necrotic myofibers (asterisk) being surrounded by extensive infiltrates of heterophils admixed with macrophages (arrowheads). **d** Ulcerative dermatitis was commonly associated with secondary colonization by bacteria (arrowheads) and fungal hyphae (arrow). **e** Severe necrotizing panuveitis with heterophilic and histiocytic inflammation expanding the choroid (asterisk) and extending into the sclera (S) and infiltrating the episclera. **f** Severe heterophilic vitritis with the vitreous chambers (V) containing large numbers of heterophils and fewer macrophages admixed with fibrin and hemorrhage

In fish challenged with LMBV by IP or by immersion, multifocal hepatic necrosis, multifocal splenic necrosis, multifocal necrotizing steatitis, and to a lesser extent, necrotizing nephritis were the most dominant lesions observed (Fig. 6). Early lesions in the liver included foci of coagulative necrosis characterized by loss of nuclear detail and hypereosinophilia of affected hepatocytes. Lesions expanded quickly involving large portions of the hepatic parenchyma with hepatocytes in affected areas undergoing degeneration and necrosis as evidenced by cytoplasmic vacuolation, nuclear pyknosis and ultimately, accumulation of cellular debris admixed with infiltrating heterophils (Fig. 6a, b). Lesions in the spleen were similar, starting as focal areas of necrosis and lymphocytolysis characterized by foci of karyorhectic debris (Fig. 6c) that quickly expanded to confluent areas of coagulative necrosis (Fig. 6d). Necrotizing steatitis, especially affecting the mesenteric fat, was a consistent lesion in all fish regardless of the route of inoculation and presented as focal areas of necrosis as evidenced by nuclear pyknosis and accumulation of karyorhectic debris surrounded by infiltrating heterophils (Fig. 6e). Necrotic adipocytes commonly underwent saponification and were surrounded by large numbers of macrophages. Focal areas of fibrinoid necrosis were observed in the kidneys of some fish and in those animals renal tubules were focally lost and replaced by accumulation of fibrin admixed with cellular debris and macrophages (Fig. 6f). In one fish challenged with the IP route, necrotizing myocarditis was observed. Only fish in the immersion group developed severe skin lesions characterized by severe epithelial necrosis that lead to focally extensive ulcerative dermatitis (Fig. 7a). The affected epithelium was characterized by cytoplasmic swelling and vacuolation of epithelial cells, nuclear pyknosis and ultimately cellular necrosis (Fig. 7b). Severe inflammation extended into the underlying muscle causing massive myofiber necrosis (Fig. 7c). Necrotic myofibers were surrounded by extensive infiltrates of heterophils admixed with macrophages. The necrotizing dermatitis and myositis locally progressed to severe heterophilic and granulomatous inflammation associated with secondary colonization by bacteria and fungal hyphae (Fig. 7d). Exophthalmia observed in SMB challenged via the immersion route and the affected eye had a severe necrotizing panuveitis and keratinitis. There were marked necrosis and heterophilic and histiocytic inflammation expanding the uveal tract including the iris, the ciliary body, and the choroid (Fig. 7e) and causing secondary vitritis (Fig. 7f). The uveal tract also contains multifocal hemorrhage and fibrin, and inflammatory infiltrates fill the ciliary cleft, obscure the trabecular meshwork and extend into the limbus and sclera. Inflammatory cells also dissect underneath Descemet’s membrane, commonly extended into the cornea causing perforating ulcers and severe conjunctivitis and infiltrate the episclera. All chambers of the eye contain fibrin and hemorrhage admixed with large numbers of heterophils and fewer macrophages.

### Phylogeny of LMBV isolated from SMB mortality episodes

BLAST analysis showed that the sequences from SMB-LMBV are identical to the sequence from LMB-LMBV (FR682503). BLAST analysis also showed that SMB-LMBV sequences shared 99.07% (1379/1392 bp) sequence identity with the two LMBUSV sequences from fish in China and Thailand that were associated with ulcerative lesions (GU256635 and KU507315).

### Effect of temperature on LMBV pathogenicity

**Fig. 8:**
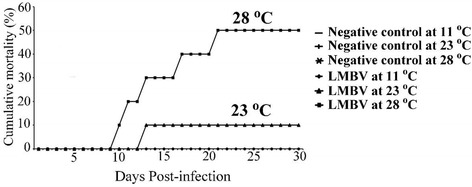
Effects of water temperature on smallmouth bass mortality. Effects of water temperature on mortality in smallmouth bass (*Micropterus dolomieu*) challenged with largemouth bass virus

SMB in the negative control group exhibited no clinical signs or mortality and were free of LMBV regardless of temperature. No mortality or clinical signs were observed in LMBV-infected SMB at a water temperature of 11 °C. At a water temperature of 23 °C, one fish died on day 13 pi, resulting in a cumulative mortality of 10% and a mean survival time (using 30 days as the upper limit for calculations) of 28.3 days (SE = 1.6 days). Log-rank and Fisher’s exact tests indicated that differences in Kaplan-Meier survival curves and cumulative mortality between the LMBV and control treatments were not statistically significant at this temperature (log-rank test: Chi-square = 1.0, *P-*value = 0.317; Fisher exact test: *P-*value = 0.5). When SMB were immersed in LMBV-tainted water at a concentration of 10^2.79^ TCID50/ml and kept at 28 °C, 50% of the fish died with a mean survival time of 22.2 days (SE = 2.6 days) (Fig. 8). Log-rank and Fisher’s exact test indicated that differences in Kaplan-Meier survival curves and cumulative mortality between the LMBV and control treatments were statistically significant at this temperature (log-rank test: Chi-square = 6.5, *P-*value = 0.012; Fisher exact test: *P-*value = 0.032).

Gross pathological changes of dead and moribund SMB maintained at 23 and 28 °C were similar to those observed in infected SMB via the immersion route. LMBV was re-isolated and confirmed from tissue samples from all dead and surviving SMB in the LMBV treatment group that were maintained at 23 and 28 °C. However, LMBV was not isolated or detected in tissues by PCR in any of the surviving fish from the LMBV treatment group that were maintained at 11 °C.

### Effect of co-infection by *F. Columnare* on LMBV-infected SMB

**Fig. 9:**
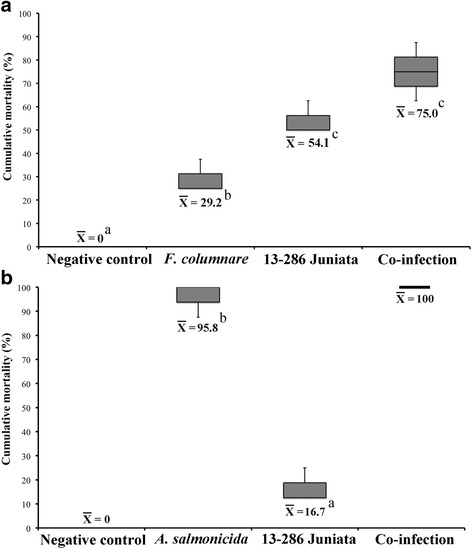
Effects of co-infecting largemouth bass virus (LMBV)-infected smallmouth bass (*Micropterus dolomieu*) with bacteria: **a** *Flavobacterium columnare*. Both experimental infections were performed at a water temperature of 28 °C. **b** *Aeromonas salmonicida.* Experimental infection with LMBV took place at a water temperature of 28 °C, while that of *A. salmonicida* was performed when the water temperature dropped to 20 °C. Data are displayed as box plots of cumulative mortality (%) displaying the median and upper/lower quartiles; whiskers indicate the maximum and minimum values in each group. Different superscript letters indicate significant difference (*P* < 0.05)

There was an overall significant difference in cumulative mortality between the treatments (*F*_3,8_ = 12.03; *P*-value = 0.002). Cumulative mortality for the control treatment was 0.0%. For the LMBV treatment, cumulative mortality was 54.1% (SE = 5.2%). For the *F. columnare* treatment*,* cumulative mortality was 29.2 (SE = 4.7%). For the co-infection treatment, cumulative mortality was 75.0% (SE = 4.5%) (Fig. 9a). Pairwise comparisons indicated that cumulative mortality in the LMBV and co-infection treatments were significantly greater than in the *F. columnare* treatment (*F. columnare* vs LMBV: − 3.41, df = 8, *P-*value = 0.028; *F. columnare* vs co-infection: − 5.99, df = 8, *P-*value = 0.001); however, the pairwise comparison between the LMBV and co-infection treatments were not significantly different (LMBV vs co-infection: − 2.93, df = 8, *P-*value = 0.057).

There were no significant differences in Kaplan-Meir survival curves among the replicates for any of the treatments based on log-rank tests. However, there was a significant difference in Kaplan-Meier survival curves among the treatments (log-rank test: Chi-square = 31.8, *P*-value< 0.001). For SMB infected with LMBV, mean survival time (using 30 days as the upper limit for calculations) was 21.7 days (SE = 1.7 days). For SMB infected with *F. columnare*, mean survival time was 25.2 days (SE = 1.6 days). When LMBV-infected SMB were subsequently infected with *F. columnare*, mean survival time was 17.5 days (SE = 0.7 days). Pairwise comparisons of Kaplan-Meir survival curves by log-rank tests indicated that survival curve differences between the *F. columnare* and co-infection treatments were significantly different (*P-*value = 0.005). Pairwise comparisons between the *F. columnare* and LMBV treatments (*P-*value = 0.314) and the LMBV and co-infection treatments (*P*-value = 0.279) were not significantly different.

No gross pathology or clinical signs were observed in the mock-infected SMB. LMBV was recovered from the tissues of kidneys, spleen and liver of all survivors in the LMBV and co-infection treatments. No *F. columnare* was recovered from any SMB that survived the bacterial infection. *F. columnare* and LMBV were re-isolated from the moribund and dead SMB co-infection treatments group and their identities were confirmed by PCR. External and internal clinical signs of dead/moribund and surviving LMBV-infected SMB were consistent with those described above. Immersion of *F. columnare* alone resulted in skin discoloration, subdermal hemorrhage, abdominal distension, and fin erosion and ulceration. Internally, organs appeared edematous with hemorrhage within kidneys, hepatomegaly, and splenomegaly.

### Effect of co-infection by *A. salmonicida* on LMBV-infected SMB

There was an overall significant difference in cumulative mortality between the treatments (*F*_3,8_ = 12.03; *P*-value = 0.002). Cumulative mortality for the control treatment was 0.0%. For the LMBV treatment, cumulative mortality was 16.7% (SE = 4.4%). For the *A. salmonicida* treatment*,* cumulative mortality was 95.8 (SE = 2.4%). For the co-infection treatment, cumulative mortality was 100.0% (Fig. 9b). Pairwise comparisons indicated that cumulative mortality in the LMBV treatment was significantly lower than in the *A. salmonicida* treatment (*A. salmonicida* vs LMBV: *t* = − 7.06, df = 8, *P-*value = 0.003). Pairwise comparisons of the *A. salmonicida* and LMBV treatments with the co-infection treatment were not estimable presumably because of the lack of variability in cumulative mortality results for the co-infection treatment replicates. Even though this prevents us from attaching statistical significance, we believe there is clear biological significance between the co-infection treatment cumulative mortality of 100% and the LMBV treatment cumulative mortality of 16.7%.

There were no significant differences in Kaplan-Meir survival curves among the replicates for any of the treatments based on log-rank tests. There was a significant difference in Kaplan-Meier survival curves among the treatments (log-rank test: Chi-square = 89.6, *P*-value< 0.001). For SMB infected with LMBV, mean survival time (using 30 days as the upper limit for calculations) was 27.3 days (SE = 1.4 days). For SMB infected with *A. salmonicida*, mean survival time was 13.8 days (SE = 0.7 days). When LMBV-infected SMB were subsequently infected with *A. salmonicida*, mean survival time was 14.8 days (SE = 0.8 days). Pairwise comparisons of Kaplan-Meir survival curves by log-rank tests indicated that survival curve differences between the *A. salmonicida* and LMBV treatments were significantly different (*P-*value< 0.001). Differences between the LMBV and co-infections treatments were also significantly different (*P*-value< 0.001). There were not significant differences in the survival curves between the *A. salmonicida* and co-infection treatments (*P-*value = 1.000).

Gross signs in SMB exposed to *A. salmonicida* only were more severe when compared to *F. columnare* infections (e.g. dermal hemorrhage, ulceration and necrosis on skin, petechial hemorrhage and hyperplasia of gill filaments, hemorrhage on base of all fins along with severe fin erosion and abdominal distension). Internally, ascites, hemorrhage of liver, splenic enlargement and darkening, and distended gastro intestine containing yellowish mucoid content were observed. Kidneys appeared edematous and hemorrhagic.

## Discussion

The findings of this study demonstrate that LMBV infection, regardless of the method of exposure, is lethal to YOY SMB. Pairwise sequence analysis based on full-length major capsid protein gene nucleotide sequences demonstrated that the SMB-LMBV isolates were all LMBV strains identical to those isolated from LMB and different from LMBV isolates from fish in China and Thailand that were associated with ulcerative lesions. This finding reveals the ability of LMBV to cause dermal lesions, a pathology not previously reported for this virus.

The disease in the IP-infected SMB group ran an acute course with most mortalities happening within one week pi, which coincides well with disease progression described for LMBV in LMB [6]. Infection by immersion seems to have a prolonged time-to-death as most of the fish died within 2 weeks pi, reaching up to 100% mortality in SMB exposed to 10^6^ and 10^7^ TCID_50_/ml. In contrast, exposure of LMB to 10^6.5^ TCID_50_/ml LMBV by immersion only caused 17% mortality within the same observation period [6]. The fact that LMBV could be re-isolated from apparently healthy SMB that survived experimental infection for up to 5 weeks raises concerns that fish that survives LMBV infection can spread the virus to other geographical locations for perhaps prolonged time periods.

Similar to LMB, water temperature plays an important role in LMBV pathogenicity for SMB under controlled experimental conditions. Raising the water temperature above 23 °C was necessary for successful experimental infection of SMB with LMBV. Grant et al. [11] reported that an optimal temperature of 25 to 30 °C was necessary for LMBV to cause mortality in LMB, which agrees with our observations in SMB. Similar observations have been made during in vitro studies where LMBV was found to replicate in FHM and BF-2 cell lines at optimal incubation temperatures between 25 and 30 °C [11, 24]. This coincides well with the water temperatures recorded during the YOY SMB mortality episodes that fluctuated between 22 and 34 °C. High water temperatures are often associated with other stressors including low dissolved oxygen concentrations. These factors favor LMBV replication and can compromise host defense mechanisms.

In both SMB groups exposed to LMBV by either IP injection or by immersion, multifocal necrotizing steatitis/peritonitis, hepatitis and splenitis were the dominant lesions observed. Necrotizing steatitis/peritonitis as well as necrosis in the liver and spleen have been reported as consistent findings in juvenile LMB experimentally inoculated with LMBV via IP injection [7]. Steatitis/peritonitis and liver necrosis were also reported in another study of juvenile LMBV inoculated with 3 genetically different LMBV isolates [25]. However, it seems that LMBV can also induce local changes at the initial site(s) of contact with the host. For example, small, inflamed, necrotic lesions developed in the skin and muscle at the site of LMBV injection in LMB [6, 7, 26], but not at other skin locations. In contrast, in our study SMB infected by immersion developed multiple ulcerative skin and ocular lesions that were not seen in IP infected SMB. It is apparent that LMBV has tropism to cell types of different origins and that the route of infection can cause different pathology. Skin lesions independent of local injection sites, as observed in SMB infected by immersion, have never been reported in the case of LMB in the USA or Europe, but have been reported in Asia [8, 9]. It is noteworthy, however, that the two Asian strains are phylogenetically distinct when compared with the LMBV isolates of this study. A similar disease course has been described for other ranaviruses (e.g., ranavirus 3) in several amphibian species, where lesions ranged from ulcerative dermatitis to wide spread necrosis in parenchymal organs [27].

Based on the gross and microscopical lesions, it is possible that the dermal lesions observed in SMB in affected rivers are initiated primarily by LMBV and that affected areas became colonized by opportunistic bacteria and fungi; both of which are abundant in the aquatic environment. It is also possible that the rapidly developing LMBV-induced necrotic changes in hematopoietic tissue may have compromised the host defense mechanisms leading to secondary infections. The wide tissue distribution of LMBV in the infected SMB and variation of lesions based on route of infection are highly suggestive that different routes of infection are effective for transmitting disease. These factors combined can lead to explosive propagation of a deadly virus among a naïve susceptible population, and can lead to die offs of the magnitude reported in the affected rivers.

One alternative explanation is that bacteria known to cause lethal disease outbreaks and associated with dermal lesions at warmer water temperatures, such as *F. columnare* or motile *Aeromonas* spp. [28], might be the primary cause of YOY SMB mortality without the involvement of LMBV. While this hypothesis is plausible, no bacterium has consistently been isolated from affected SMB. On the contrary, our study demonstrated that *F. columnare* is more likely a contributor to overall SMB mortality in natural disease outbreaks. In our study, SMB infected with *F. columnare* alone developed skin lesions and mortalities at a much lower magnitude compared to LMBV-infected SMB, despite large numbers of bacteria having been added to the water at environmentally unrealistic levels (~ 10^8^ cfu/ml). Instead, co-infection of LMBV-infected SMB with *F. columnare* significantly elevated mortalities. This finding further supports the hypothesis that SMB are either immunocompromised through infection with LMBV or with a damaged skin barrier due to viral infection, and can therefore easily contract secondary bacterial infections.

At the same water temperature at which LMBV and *F. columnare* were able to infect SMB causing dermal lesions, *A. salmonicida*, when added to the water at a concentration of 10^8^ cfu/ml, was unable to cause mortalities or lesions in SMB (data not shown). It is therefore unlikely that *A. salmonicida* plays a causative role in SMB mortalities during the summer months. In contrast to LMBV, *A. salmonicida* was highly pathogenic to SMB at a lower temperature, 20 °C, at which LMBV pathogenicity was substantially diminished. Our findings clearly demonstrate that *A. salmonicida* alone can cause high mortality associated with dermal lesions albeit at much lower water temperature.

## Conclusions

Co-infection of LMBV with *F. columnare* or *A. salmonicida* may have important implications for YOY SMB mortality across a wide temperature range. During warmer summer months, LMBV alone or in combination with *F. columnare* can cause YOY SMB mortality at the magnitude noticed in the multiple river systems. As discussed earlier, *F. columnare* alone cannot be the primary cause of mortality since, even at extremely high concentrations in experimental settings, it does not cause mortality levels as high as reported for the Susquehanna and Potomac River basins. In addition, since *F. columnare* has a wide host range, other fish species should have been affected as well. In contrast, LMBV has a much narrower host range and primarily infects centrarchids [6, 7, 27]. While at lower water temperatures, certain bacterial pathogens such as *A. salmonicida* could become a major contributor to mortality episodes, though their host range is also wide and such episodes of high mortality should have involved other fish species as well. The observed YOY SMB fish kills in PA occur at warmer temperatures and as a result most likely are not the result of an *A. salmonicida*, however the results of this study show that the pathogen could play a role in SMB fish kills in other river systems or under different conditions.

The sum of the data generated in this study, including LMBV ability to cause, without co-infection, high mortality rates associated with dermal lesions under laboratory conditions that are visually similar to the skin lesions observed in YOY SMB during the mortality episodes, and its relatively high optimal temperature which coincides with those prevailing at the affected rivers during the peak of mortality episodes, strongly suggest that this iridovirus is the most likely primary cause of this large-scale mortality afflicting SMB, a local recreationally and economically important fish. While chemicals and other adverse environmental factors could be indirectly involved (e.g., immune suppressor), their role in the mortality episodes remains unclear.

## Abbreviations

BLAST: Basic local alignment search tool
CPE: Cytopathic effect
DFV: Doctor fish virus
FHM: Fathead minnow cell line
IACUC: Institutional Animal Care and Use Committee
IP: Intraperitoneal
LBUSV: Largemouth bass ulcerative syndrome virus
LD_50_: Median lethal dose
LMB: Largemouth bass
LMBV: Largemouth bass virus
MCP: Major capsid protein gene
MSU: Michigan State University
PBS: Phosphate buffered saline
SMB: Smallmouth bass
TCID_50_: Tissue culture infectious dose
TCM: Tissue culture medium
YOY: Young of the year
